# The effects of prior exposure to prism lenses on *de novo* motor skill learning

**DOI:** 10.1371/journal.pone.0292518

**Published:** 2023-10-20

**Authors:** Annmarie M. Lang-Hodge, Dylan F. Cooke, Daniel S. Marigold

**Affiliations:** 1 Biomedical Physiology and Kinesiology, Simon Fraser University, Burnaby, British Columbia, Canada; 2 Institute for Neuroscience and Neurotechnology, Simon Fraser University, Burnaby, British Columbia, Canada; Universite Catholique de Louvain, BELGIUM

## Abstract

Motor learning involves plasticity in a network of brain areas across the cortex and cerebellum. Such traces of learning have the potential to affect subsequent learning of other tasks. In some cases, prior learning can interfere with subsequent learning, but it may be possible to potentiate learning of one task with a prior task if they are sufficiently different. Because prism adaptation involves extensive neuroplasticity, we reasoned that the elevated excitability of neurons could increase their readiness to undergo structural changes, and in turn, create an optimal state for learning a subsequent task. We tested this idea, selecting two different forms of learning tasks, asking whether exposure to a sensorimotor adaptation task can improve subsequent *de novo* motor skill learning. Participants first learned a new visuomotor mapping induced by prism glasses in which prism strength varied trial-to-trial. Immediately after and the next day, we tested participants on a mirror tracing task, a form of *de novo* skill learning. Prism-trained and control participants both learned the mirror tracing task, with similar reductions in error and increases in distance traced. Both groups also showed evidence of offline performance gains between the end of day 1 and the start of day 2. However, we did not detect differences between groups. Overall, our results do not support the idea that prism adaptation learning can potentiate subsequent *de novo* learning. We discuss factors that may have contributed to this result.

## Introduction

Motor learning is an experience-dependent improvement in motor behavior and occurs throughout life [1]. Uncovering methods to enhance this learning, whether through speeding up initial performance improvements, increasing overall performance, or extending the retention of a specific performance level, is important. This is because some form of motor learning is necessary for recovering function or compensation following neurological and musculoskeletal injury, to excel in athletics, and for vocational tasks.

One form of motor learning, sensorimotor adaptation, is studied with visuomotor perturbation paradigms like prism adaptation or visuomotor rotation. This learning involves a rapid, explicit, aiming-strategy component [2] and a slower, implicit, sensory-prediction-error-driven mechanism [3, 4]. The explicit component (also known as strategic control) refers to recalibrating sensorimotor reference frames coding target location and strategically aiming to hit a target; this component contributes mainly to the early phase of adaptation [5, 6]. The implicit mechanism (also known as spatial realignment) refers to adjusting visuomotor and/or proprioceptive-motor mappings (updating an internal model) by minimizing sensory prediction errors arising from a mismatch between actual sensory feedback and predicted feedback generated from an internal forward model [3, 5, 6]. This process contributes more to later phases of adaptation. Adaptation is characterized by the presence of aftereffects when the perturbation is removed or the participant attempts a different motor task, which likely reflect updating of an internal model [5–8]. A memory of this adaptation can become resistant to opposite perturbations [7–9] and can last for extended periods of time (>1 year) [10, 11].

*De novo* skill learning represents another form of motor learning. Rather than adapting an existing control policy as with prism adaptation, *de novo* learning involves creating a new control policy [1, 12–14]. Mirror-reversal tasks are examples of *de novo* learning [13, 14]. Mirror-reversal and other motor skill learning tasks are characterized by improvements in performance with practice and distinguished from adaptation tasks by the presence of offline gains, i.e., performance improvements at the start of the next session relative to the end of the initial learning phase [13, 15].

A potential way to facilitate motor learning is to prime the brain before learning begins [16, 17]. That is, increasing the readiness of relevant neural circuitry to undergo plastic changes. In the present study, we asked whether priming the brain with adaptation learning can improve subsequent *de novo* motor skill learning. We chose prism adaptation, which is well known to induce neuroplasticity across several brain regions. For example, prism adaptation causes activation of several regions within the posterior parietal cortex (PPC) and cerebellum [18], alters post-adaptation resting-state functional connectivity in frontoparietal and cerebellar-parieto-parahippocampal networks [19], and is improved and better retained when paired with transcranial direct current stimulation over the primary motor cortex, or simultaneously over the PPC and cerebellum [20, 21]. Furthermore, prism adaptation may increase motor cortex excitability, as reflected by greater motor evoked potential mean amplitude and a steeper slope of the input-output curve following transcranial magnetic stimulation [22, 23]. Prism-reaching studies in monkeys also show persistent changes in PPC single neuron tuning and overall firing rate [24].

We predicted that adapting to prisms in one motor task would increase the excitability of neurons and their readiness to undergo structural changes, and in turn, create an optimal state for learning a subsequent task. In this sense, having to adapt to prisms would prime the brain and facilitate learning like neuromodulation techniques do [25, 26]. This could provide a non-stimulation-based approach to facilitating learning and recovery of function following injury. Previous research demonstrates that prior exposure to visuomotor rotations (which also alter visuomotor mappings like prisms) leads to faster sequence learning and adaptation to reaching movements with increased cursor gain [17], providing support for the idea that one form of adaptation learning may facilitate other forms of learning. Moreover, prism adaptation may impact reinforcement learning. Left- (but not right-) shifting prisms increase reward-based learning [27], which may relate to asymmetries in dopamine receptors [28]. As we sought to test the effects of elevated neural activity in a broad array of potential brain regions on motor learning, this study is not directly relevant, but it does indicate one more way in which one form of learning might affect others.

For the *de novo* motor skill task, we chose mirror-reversal tracing. Motor skill learning, in general, involves changes in several brain regions, including the motor cortex [29], basal ganglia [30], and cerebellum [31]. Recently, Kodama et al. [32] showed significant increases in MRI-detected gray matter in the primary motor and somatosensory cortices and hippocampus after the first day of mirror-reversal learning, which predicted performance on later learning. Although little is known about the neural basis of mirror tracing, the PPC is also potentially involved. This is because the visuomotor mapping is altered in mirror tracing, and PPC is active when learning new visuomotor mappings, regardless of whether it is during a prism adaptation or visuomotor rotation paradigm [18, 33]. Thus, there is likely an overlapping network of brain areas (e.g., PPC, cerebellum, motor cortex) involved in both prism adaptation and mirror tracing.

We therefore tested whether acquisition of a new visuomotor mapping induced by prism glasses can improve learning of a mirror-tracing task. We had one group of participants adapt to prisms during a seated pointing task, followed by learning a new mirror-tracing task. We had another group perform the seated pointing task with blank lenses prior to learning the mirror-tracing task. We tested both groups a second time on the tracing task the following day. In the prism adaptation paradigm, we varied prism strength from trial to trial, which masked the mean prism shift (18-diopters) and is known to slow the rate of adaptation (but not so much that adaption does not occur within 60 trials) [3]. The varying prism strengths increase task difficulty and cause the brain to have to adapt for longer than a simple constant perturbation. Varying the prism shift from trial-to-trial could affect explicit aiming strategies (i.e., strategic control), as the aiming strategy for one trial would not appropriately counter the prism shift in a subsequent trial (at least until the mean prism shift is learned). It may also affect implicit internal model updating (i.e., spatial realignment). Specifically, the randomly changing prism strengths reduce the certainty with which the brain can use visual feedback about a given movement error to estimate the difference between its current estimate of the visuomotor mapping and the mapping (in our case, the mean prism shift) required to hit a target. Consequently, the state estimate that is necessary to plan the motor command is less affected by prism-induced sensory prediction errors and instead, relies more on forward model predictions [3]. Both effects contribute to slow the rate of adaptation. In addition, the prolonged exposure to larger errors and challenge of determining the appropriate visuomotor mapping (presumably the mean prism shift) means that the brain must continuously adjust weightings of neural circuitry until it learns to counter the prisms. Taken together, these factors may contribute to slower adaptation (error reduction) previously observed [3], which we hypothesize could increase plasticity and learning of other tasks.

## Methods

### Participants

Twenty-five participants (age 22.7 ± 3.0; 11 men, 14 women; all right-hand dominant) from Simon Fraser University and the surrounding area participated in this study between July and November 2022, inclusive. We did not perform any a priori power analysis to determine sample size. Instead, we used group sample sizes typical in the literature for this type of research (for example, [7, 14, 34–36]). Participants had no known visual diseases or neurological or musculoskeletal disorders impairing movement. No participants had previously participated in a research study using prism glasses. The Office of Research Ethics at Simon Fraser University approved the study protocol, and all participants provided written informed consent before starting the experiment.

### Experimental tasks and protocol

**Fig 1:**
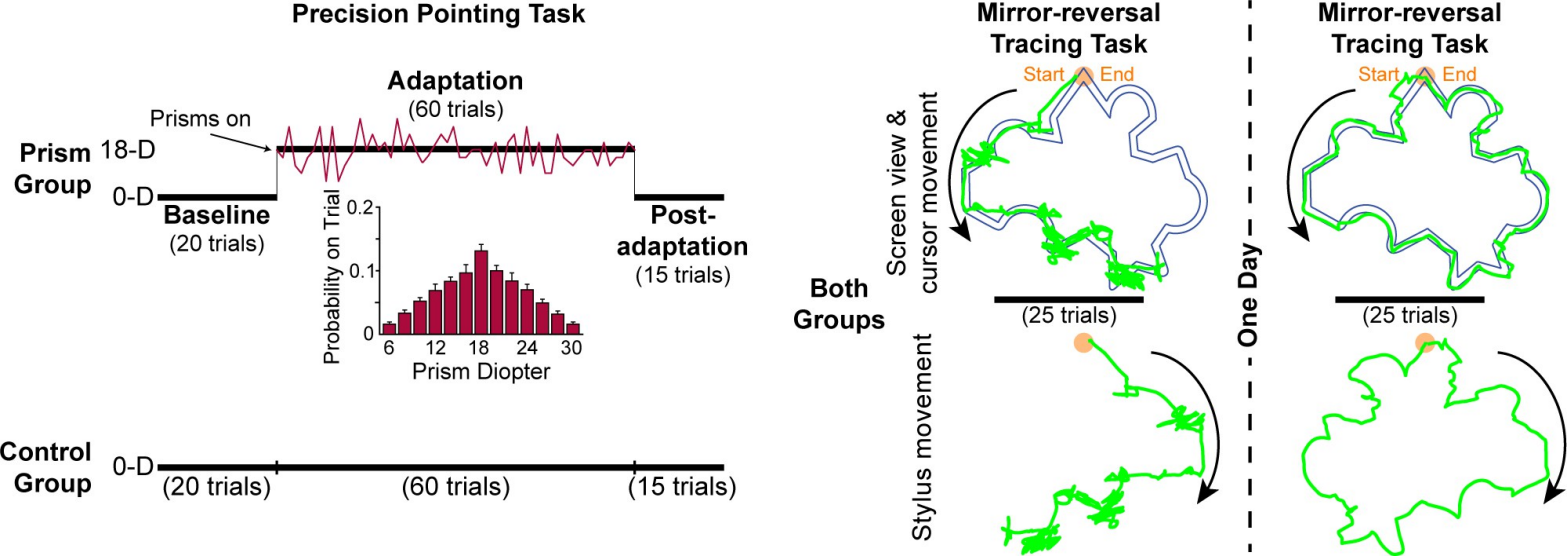
Experimental tasks and protocol. Both groups first performed 95 trials of a precision pointing task from a seated position. The control group wore goggles with 0-diopter (0-D) lenses that did not alter visual perception. The prism group wore goggles with varying strength, rightward-shifting prism lenses (mean strength = 18-diopters, 18-D; distribution of lens strengths shown) for 60 trials after pointing with 0-diopter lenses for 20 trials. This group pointed for an additional 15 trials while wearing 0-diopters lenses. Participants in both groups had vision of the target and hand during the pointing movements in all phases (i.e., closed-loop pointing). Both groups then performed 25 trials of a tracing task in which we reversed the relationship between the stylus and cursor motion in the left-right direction, such that a rightward movement of the stylus would cause the cursor on the computer screen to move leftward. The following day, both groups repeated the tracing task. At right are examples of tracing performance (green) from one participant on trial 1 of day 1 (left) and trial 1 of day 2 (right). Top, screen view seen by the participant (colors have been changed here for clarity) with counterclockwise cursor path superimposed on the target shape (166 mm wide x 152 mm tall; path = 5 mm wide). Bottom, clockwise stylus movement. Orange circle at top of shape is the tracing start and end point.

All participants performed a precision pointing task followed by a mirror-reversal tracing task, both being completed within ~70 minutes. The next day, participants returned to the laboratory and performed the identical mirror-reversal tracing task again. Participants used their dominant (right) hand for each task. Details are illustrated in Fig 1.

For the precision pointing task, participants sat with a target (vertical length = 12 cm; width = 1 cm) positioned at eye level and at a comfortable reaching distance (approximately arm’s length) in front. Participants wore goggles to house removeable lenses (see below), and kept their eyes closed except when pointing to the target. We instructed participants to reach from their chin to the target as quickly and accurately as possible, aiming for the medial-lateral (ML) center of the target. These instructions helped to reduce the possibility of online corrections of the finger during the pointing movement. We asked participants to hold their finger on the target for one second before bringing it back to their chin. An experimenter provided a verbal cue to denote the start of a trial, at which time, the participant opened their eyes to perform the movement. The vertical length of the target minimized the need for accuracy in the vertical direction because the prisms (see below) cause errors in the ML direction and our focus was ML error. An Optotrak Certus motion capture camera (Northern Digital, Waterloo, ON, Canada), placed perpendicular to the screen, recorded an infrared-emitting position marker on the medial side of the right index finger, near the tip, at a sampling frequency of 100 Hz.

We randomly assigned participants to a prism group (n = 13) or a control group (n = 12). We did not inform participants about the prism shift. Two participants in the prism group did not follow instructions, one during the pointing task and the other during the tracing task. Specifically, in the pointing task, the participant did not pay attention and did not open and close their eyes as instructed. In the tracing task, the participant drew the shape as they saw it (not mirror-reversed) and did not attempt to keep within the shape’s lines. Thus, we excluded these individuals from all analyses, which left 11 in the prism group and 12 in the control group. The research team was not blinded to participant group assignment given the nature of the tasks. Participants in both groups had vision of the target and vision of the hand during the pointing movements (i.e., closed-loop pointing), except for a brief initial movement period in each trial due to the construction of the goggles. All participants initially performed 20 baseline trials while wearing goggles with 0-diopter lenses. The prism group then performed 60 trials while wearing goggles with rightward-shifting prism lenses that induced a new visuomotor mapping. We used a noise paradigm [3] to vary the strength of the prism lenses for each trial based on a Gaussian distribution (see Fig 1) with a mean of 18-diopters (10.3°) and range of 6- to 30-diopters (2-diopter increments). The order of prism strengths was random except for the first and last adaptation trial, which we set to 18-diopter. To mask the strength of the prism shift, an experimenter removed and replaced the prism lenses from the goggles every trial. The control group performed 60 trials after their baseline trials wearing 0-diopter lenses, which we removed and replaced between each trial to match the procedure for the prism group. Both groups finished with 15 pointing trials with 0-diopter lenses. For the prism group, these trials (post-adaptation phase) enabled us to washout any potential anterograde interference effects of the prisms on the tracing task [9, 37, 38]. Anterograde interference occurs when learning of one visuomotor mapping disrupts initial learning of a subsequent (different or opposite) mapping, which would potentially mask true initial learning on the latter task (in our case, mirror-reversal tracing). Pilot testing without these washout trials suggested that anterograde interference impacted early tracing performance.

After completing the precision pointing task, all participants performed 25 trials of a mirror-reversal tracing task, starting ~3–5 minutes after the last pointing trial. We did not inform participants about the mirror-reversal. In this task, participants sat in front of a laptop computer screen and used their dominant hand to trace between two lines forming a path around the perimeter of a shape with a stylus and tablet (Wacom Intuos Pro, medium, PTH660). We set the tablet and computer 5 cm and 31.5 cm from the edge of the table, respectively. Using Adobe Illustrator (Adobe Inc., San Jose, CA, USA), we designed an irregular shape to trace, which we imported into MATLAB (The MathWorks, Natick, MA). It comprised four semi-circles and 10 pairs of lines set at varying angles. A circle at the top of the shape marked the start and end point (see Fig 1). The width of the shape’s path measured 5 mm on the screen and 3.5 mm on the tablet. The maximum height of the shape on the screen measured 15.2 cm and the maximum width measured 16.6 cm. A custom-written MATLAB program with the Psychophysics Toolbox, version 3 [39] collected the tracing data. To generate the mirror reversal, we reversed the sign of the x position (left-right direction) of the cursor. We instructed participants to trace the shape, moving their hand to the right first, as quickly and accurately as possible in a 90-s time frame. In addition, we instructed participants to maintain gaze on the computer screen while tracing and not to lift the stylus off the pad, as it would lose the position in the shape. We eliminated a trial if the participant moved their hand to the left initially (0.7% of trials) or if the computer did not register the stylus on the tablet (1.2% of trials). A timer in the top left corner of the screen allowed the participant and researcher to see how much time had elapsed in each trial. If they finished before 90 s, we had them keep the stylus on the start/end point and press a key to proceed to the next trial. There were no inter-trial breaks during this task. All participants returned to the laboratory the next day to perform the same mirror-reversal tracing task again.

### Data and statistical analyses

To quantify performance on the precision pointing task, we first filtered the finger marker data using a fourth-order, low-pass Butterworth filter (cut-off frequency of 6 Hz). Next, we determined the endpoint of the pointing movement to the target, which we defined as the time at which the finger position marker’s anterior-posterior velocity profile stabilized to near zero [34]. A total of 5.1% of trials had missing kinematic data from the finger such that we could not quantify performance. Missing data were likely the result of the participant slightly rotating their finger near the end of the pointing movement such that the position marker was no longer visible to the motion capture camera. We quantified performance (i.e., ML error) as the ML distance between the finger marker at the endpoint of the movement relative to the ML center of the target. We defined a positive value as ML error to the right, in the direction of the prism shift, and a negative value as error to the left (opposite of the shift). To determine whether participants in the prism group adapted, we compared ML error during the baseline phase (mean of the last 10 trials), first adaptation trial (or corresponding trial for the control group), late adaptation (mean of the last 10 adaptation trials or corresponding trials for the control group), and the first post-adaptation trial (or corresponding trial for the control group) using a linear mixed-effects model, with Group and Phase as fixed effects and participant as a random effect. Note that we used only the first post-adaptation trial to assess aftereffects because participants start to de-adapt after this trial given that they have visual feedback during this phase.

To quantify performance on the mirror-reversal task, we first used a 5-point central moving average to filter the data. We sampled the tracing data at 100 Hz. Finally, we quantified three measures: % error, completion amount, and the number of times the trace crossed the lines of the path (normalized by the completion amount; hereafter, referred to as crossing points). Specifically, we defined % error as the percentage of time the participant spent tracing outside of the path. For completion amount, we determined the centroid of the shape and converted the tracing data to polar coordinates. Subsequently, we calculated how much of the shape the participant traced, in degrees from the start position, within the 90-s time limit.

To determine how participants learned the tracing task on day 1 and whether groups learned differently, we binned the 25 trials into five five-trial averages for each of the % error, completion amount, and crossing points measures. Next, we performed separate linear mixed-effects models for each measure, with Group and Bin as fixed effects and participant as a random effect. For the crossing points measure, we found an outlier (studentized residual of 4.7); however, removing it did not change the results and thus, we left this data point in the model. To determine the presence of offline gains with the tracing task, we compared the average of the last five trials (i.e., bin 5) on day 1 with the average of the first five trials on day 2 between groups for each of our three measures. This entailed using separate linear mixed-effects models for each measure, with Group and Day as fixed effects and participant as a random effect. For this analysis, we log-transformed the % error data to conform to the assumption of normality.

We used JMP 16 software (SAS Institute Inc., Cary, NC) and an alpha level of 0.05 for all primary statistical analyses. We conducted Tukey’s post hoc tests following a significant main effect or interaction, where appropriate. Effect sizes are reported as $\eta_{p}^{2}$.

Given the non-significant group effects for the tracing task (see Results) and relatively small sample size, we performed complementary Bayesian mixed-effects model analyses with response variables of % error, completion amount, or crossing points. For all models, we included participant as a random effect and used a Gaussian family with the identity link function. We fit models with the brm function of the *brms* package version 2.19.0 [40] using RStudio version 2023.06.1+524 (R version 4.3.1). This used a No-U-Turn Sampler (NUTS) algorithm with 4 chains (each with 2000 iterations), burn-in iterations of 1000, thinning of 1 (except for a few models where we increased chain iterations to 8000, burn-in iterations to 4000, thinning to 2). For all models, we used the default priors provided by the brm function, which are uninformative (flat) priors. We checked each model parameter to ensure adequate mixing and convergence of Markov chain Monte Carlo chains (R-hat of 1) and checked for large effective sample sizes to ensure efficiency of the sampling. In our day 1 learning analysis, we compared different models using Bayes Factor to identify the optimal fit and evaluate the significance of individual variables. We began with a baseline including only Participant’s random effect. Subsequently, we introduced models with main effects of Bin, Group, and the Group and Bin interaction, all while accounting for the Participant’s random effect. We used a similar approach for the offline gains analysis, substituting Day for Bin. Like the linear mixed-effects model, we log-transformed % error data for this analysis.

## Results

Participants in both groups performed a precision pointing task followed by a mirror-reversal tracing task. The prism group experienced rightward-shifting prism lenses of varying strengths (around a mean of 18-diopters) during a 60-trial adaptation phase of the pointing task. The control group wore 0-diopter lenses, which did not produce any perceived shift of the visual field. The next day, both groups of participants performed the identical mirror-reversal tracing task again.

### Prism adaptation

**Fig 2:**
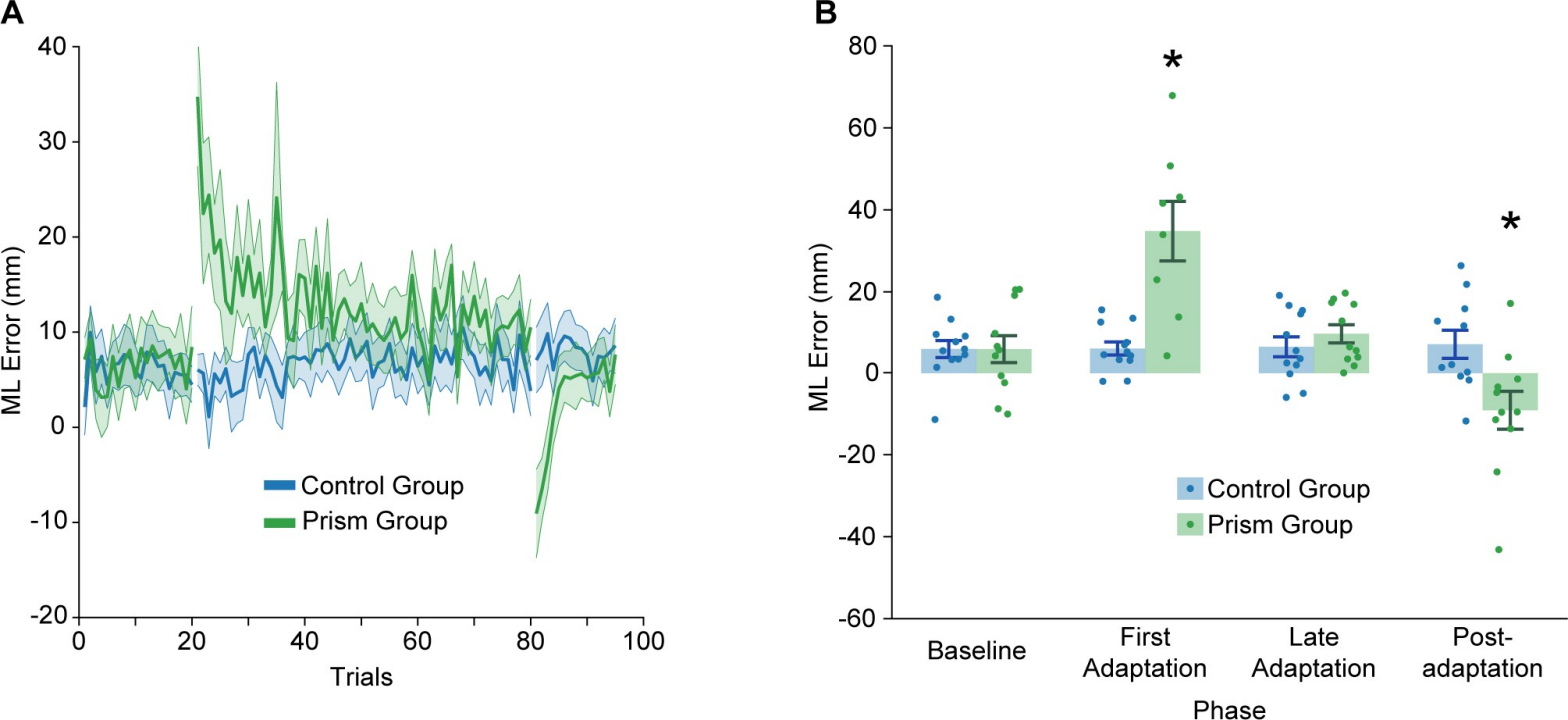
Results of the precision pointing task. **A:** Group mean ± SE, ML endpoint position error across all trials for the control (blue) and prism (green) groups. **B:** Group mean ± SE, ML endpoint position error for the baseline (or control group equivalent) phase (mean of last 10 trials), first adaptation trial (or control group equivalent), late adaptation (or control group equivalent) phase (mean of last 10 trials), and first post-adaptation trial (or control group equivalent) for the control and prism groups. For the prism group, a positive value represents errors in the direction of the prism shift. Asterisk indicates that values are significantly different from other groups/phases based on post hoc tests (p < 0.05).

Participants pointed with their right index finger to a target positioned at eye level in front of them. Fig 2A illustrates performance across trials. The control group had an ML error of 6.3 ± 8.1 mm across repeated trials. The prism group maintained a similar performance level during baseline trials and then large ML errors in the direction of the prism shift during early adaptation. Over repeated trials, the prism group reduced errors to baseline levels. Each participant had a different prism shift order (except for the first and last adaptation trial of 18 diopters), which likely explains the variability seen in the graph across trials of the adaptation phase.

To quantify group differences, we compared ML error between groups during the baseline phase (mean of the last 10 trials), first adaptation trial (or corresponding trial for the control group), late adaptation (mean of the last 10 adaptation trials or corresponding trials for the control group), and the first post-adaptation trial (or corresponding trial for the control group). We found a significant main effect of Phase (F_3,60_ = 20.0, p = 4.11e-9, $\eta_{p}^{2}$ = 0.50). We did not detect a significant main effect of Group (F_1,21_ = 1.1, p = 0.296, $\eta_{p}^{2}$ = 0.05). More importantly, we found a significant Group x Phase interaction (F_3,60_ = 21.6, p = 1.34e-9, $\eta_{p}^{2}$ = 0.52). Specifically, the prism group demonstrated significantly greater error (p < 0.05) in the first adaptation phase trial compared to the control group and other phases (Fig 2B). In addition, the prism group demonstrated ML errors in the opposite direction during the first post-adaptation phase trial, indicative of a negative aftereffect. This value differed significantly from the control group and other phases (p < 0.05).

### Mirror-reversal tracing

**Fig 3:**
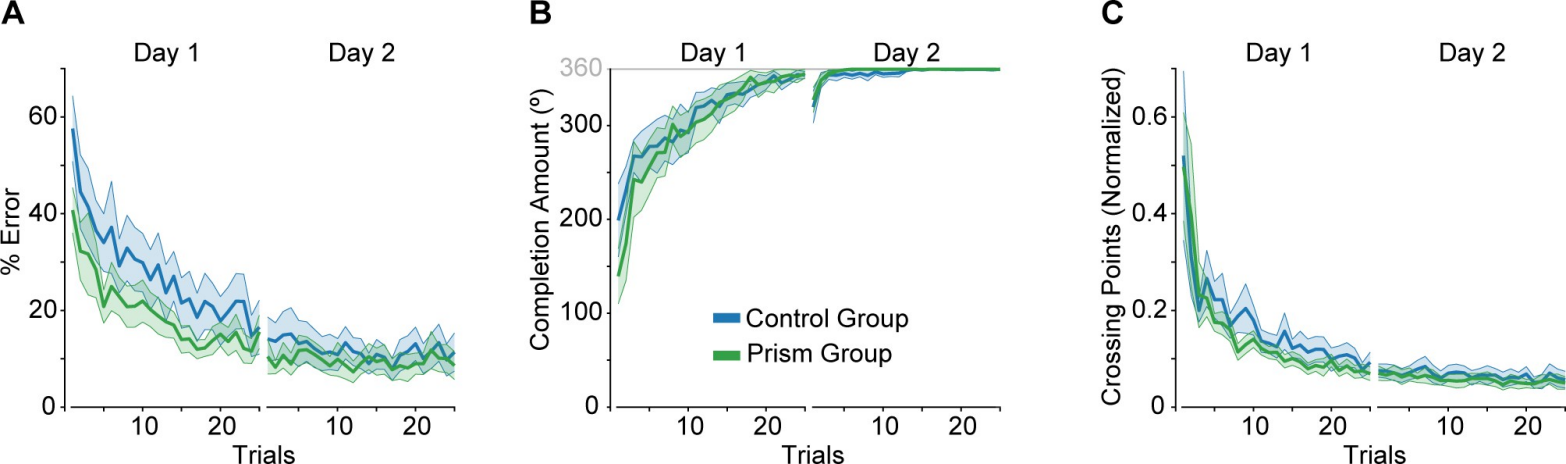
Learning profiles for the mirror-reversal tracing task. Group mean ± SE, % error (**A**), completion amount (**B**), and crossing points (**C**) across trials and days for the control (blue) and prism (green) groups.

In the tracing task, we reversed the relationship between the stylus and cursor motion in the left-right direction, such that a rightward movement of the stylus would cause the cursor on the computer screen to move leftward. Despite this perturbation, both groups of participants demonstrated improvements in performance over repeated trials. This entailed a reduction in the time spent tracing outside the path of the shape (% error, Fig 3A), an increase in the proportion of the shape traced within the allotted time limit (completion amount, Fig 3B), and a reduction in the number of times crossing the lines of the path (normalized to completion amount; Fig 3C). Both groups maintained their performance levels on day 2, though seven control and five prism group participants required at least one trial before they could complete the shape again within the allotted time (Fig 3B).

**Fig 4:**
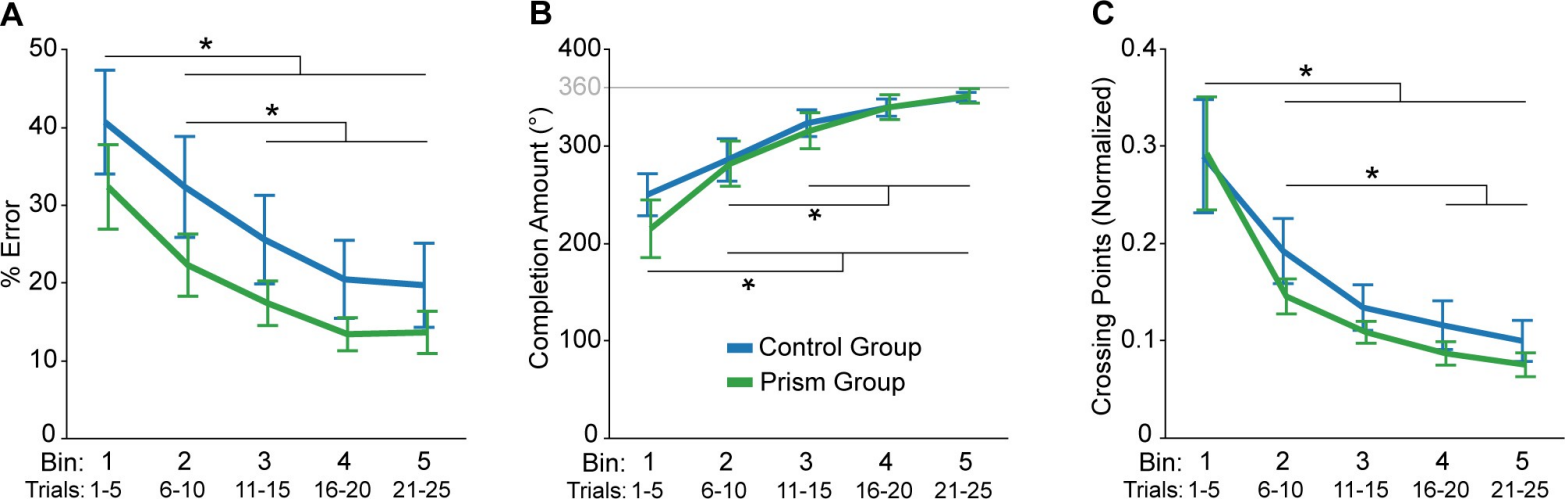
Evidence of learning on day 1 for the mirror-reversal tracing task. Group mean ± SE, % error (**A**), completion amount (**B**), and crossing points (**C**) across bins for the control (blue) and prism (green) groups. Each bin is the mean of five tracing trials for a participant, averaged within each group to produce a group mean value. Asterisk indicates that values are significantly different from each other based on post hoc tests for the main effect of bin (p < 0.05).

We first asked whether groups differed in their performance on day 1. To address this question, we compared groups across five bins (each an average of five trials) for all three of our measures. For % error, we found a significant main effect of bin (F_4,84_ = 32.8, p = 1.83e-16, $\eta_{p}^{2}$ = 0.61), with bin 1 greater than the other bins and bin 2 greater than bins 3–5 (Fig 4A). However, we did not detect a significant effect of group (Group: F_1,21_ = 1.5, p = 0.241, $\eta_{p}^{2}$ = 0.06; Group x Bin: F_4,84_ = 0.27, p = 0.899, $\eta_{p}^{2}$ = 0.01). Similarly, we found a significant main effect of bin for completion amount (F_4,84_ = 38.0, p = 3.96e-18, $\eta_{p}^{2}$ = 0.64), with bin 1 less than the other bins and bin 2 less than bins 3–5 (Fig 4B). Again, we did not detect a significant effect of group (Group: F_1,21_ = 0.18, p = 0.672, $\eta_{p}^{2}$ = 0.01; Group x Bin: F_4,84_ = 0.93, p = 0.453, $\eta_{p}^{2}$ = 0.04). In addition, we found a significant main effect of bin for crossing points (F_4,84_ = 24.4, p = 2.02e-13, $\eta_{p}^{2}$ = 0.54), with bin 1 greater than the other bins, and bin 2 greater than bins 4 and 5 (Fig 4C). We also did not detect a significant effect of group for this measure (Group: F_1,21_ = 0.50, p = 0.487, $\eta_{p}^{2}$ = 0.02; Group x Bin: F_4,84_ = 0.28, p = 0.888, $\eta_{p}^{2}$ = 0.01). Taken together, tracing performance on day 1 showed evidence of learning but did not differ between groups.

Given the non-significant group effects on day 1 learning and relatively small sample size, we performed complementary Bayesian mixed-effects model analyses with response variables of % error, completion amount, or crossing points (see S1–S3 Tables for specific details). The best fitting model for % error included participant as a random effect, both main effects (Bin and Group), and the interaction. Based on the estimates and their 95% credible intervals, we found no evidence to suggest a significant effect of Group. However, we found evidence to suggest that bins 2–5 significantly differed from bin 1 and bins 4 and 5 significantly differed from bin 2. The best fitting model for completion amount also included participant as a random effect, both main effects (Bin and Group), and the interaction. Based on the estimates and their 95% credible intervals, we found no evidence to suggest a significant effect of Group. However, we found evidence to suggest that bins 2–5 significantly differed from bin 1 and bin 5 significantly differed from bin 2. The best fitting model for crossing points included participant as a random effect and the main effect of Bin. Based on the estimates and their 95% credible intervals, we found evidence to suggest that bins 2–5 significantly differed from bin 1. Overall, these results support our primary statistical analysis.

**Fig 5:**
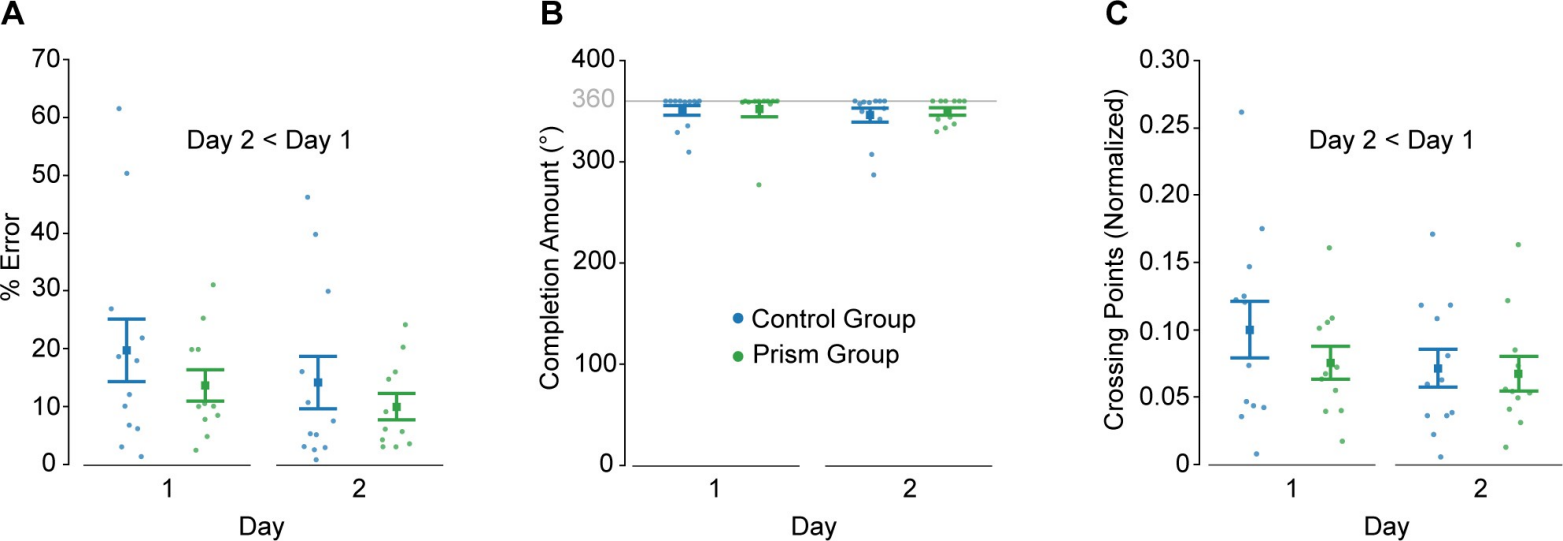
Offline gains analysis. Group mean ± SE, % error (**A**), completion amount (**B**), and crossing points (**C**) comparing the end of day 1 (mean of last five tracing trials) with the start of day 2 (mean of first five tracing trials) between groups.

We next asked whether groups differed in terms of offline gains. To address this question, we compared the average of the last five trials (i.e., bin 5) on day 1 with the average of the first five trials on day 2 between groups for all three of our measures. Both groups showed reduced % error at the start of day 2 compared to the end of day 1 (main effect of day: F_1,21_ = 21.9, p = 1.27e-4, $\eta_{p}^{2}$ = 0.51; Fig 5A), supporting the notion of offline gains. However, we did not detect an effect of group (Group: F_1,21_ = 0.02, p = 0.886, $\eta_{p}^{2}$ = 0.001; Group x Bin: F_1,21_ = 0.55, p = 0.466, $\eta_{p}^{2}$ = 0.03). As illustrated in Fig 5B, completion amount did not differ between the start of day 2 and the end of day 1 (Day: F_1,21_ = 0.93, p = 0.345, $\eta_{p}^{2}$ = 0.04) or between groups (Group: F_1,21_ = 0.1, p = 0.757, $\eta_{p}^{2}$ = 0.005; Group x Day: F_1,21_ = 0.11, p = 0.748, $\eta_{p}^{2}$ = 0.005). In fact, Fig 3B shows that, on average, both groups did not complete tracing the shape in the first two trials of day 2. This suggests that participants required some initial context to recall the motor memory of the visuomotor mapping and/or that they sacrificed tracing speed to maintain accuracy (note that % error was minimal on these day 2 trials; Fig 5A). Both groups showed reduced crossing points at the start of day 2 compared to the end of day 1 (main effect of day: F_1,21_ = 13.8, p = 0.001, $\eta_{p}^{2}$ = 0.40; Fig 5C). We did not detect a main effect of group (F_1,21_ = 0.44, p = 0.516, $\eta_{p}^{2}$ = 0.02) for this measure, but we found a trend towards a Group x Day interaction (F_1,21_ = 4.3, p = 0.052, $\eta_{p}^{2}$ = 0.17). Taken together, we found evidence of offline gains but no group differences.

Given the non-significant group effects for the offline gains analysis and relatively small sample size, we performed complementary Bayesian mixed-effects model analyses with response variables of % error, completion amount, or crossing points (see S4–S6 Tables for specific details). For % error, two models were well favored over the baseline model, one with participant as random effect and the main effect of Day, and one with participant as a random effect and both main effects of Day and Group. We selected the latter model to analyze further so that we could check for an effect of Group. Based on the estimates and 95% credible intervals, we found evidence to suggest a significant difference between days but not groups. The best fitting model for completion amount included participant as a random effect, both main effects (Day and Group), and the interaction. Based on the estimates and their 95% credible intervals, we found no evidence to suggest a significant effect of Group or Day. The best fitting model for crossing points included participant as a random effect and the main effect of Day. However, Bayes factor suggests only weak evidence in favor of this model relative to the baseline model. Overall, these results support our primary statistical analysis for % error and completion amount, though the Bayesian analysis for crossing points suggests that evidence for an effect of Day is weak.

## Discussion

The brain undergoes extensive neuroplasticity when adapting to prisms during movement. Here, we tested whether adapting to prisms could improve the subsequent learning of a mirror-reversal tracing task. We varied the prism strength from trial to trial because it slows the rate of adaptation [3], thus causing the brain to have to adapt for longer than a simple constant perturbation. We predicted that adapting to these prism shifts would increase the excitability of neurons and their readiness to undergo structural changes, and in turn, create an optimal state for learning the subsequent tracing task. This is based on the premise that many of the brain regions involved in prism adaptation, including the posterior parietal cortex, cerebellum, and motor cortex, are likely also involved in learning the tracing task. Two groups of participants performed a seated pointing task, one wearing blank lenses and the other adapting to prisms. Next, they learned a novel mirror-reversal tracing task. We tested each group again the following day on the tracing task. Both groups showed similar improvements in performance on the tracing task over repeated trials on day 1. In addition, both groups showed offline gains in terms of a reduction of the percentage of time tracing outside of the shape’s path and the number of times the trace crossed the lines of the path (normalized by shape completion amount). Our complementary Bayesian analyses largely supported these results obtained from the linear mixed-effects model analyses. Taken together, we did not find evidence that prior prism adaptation enhanced learning of the tracing task. Importantly, however, prior prism adaptation did not interfere with learning the tracing task.

Our tracing results showed evidence of offline gains and persistent steady-state accuracy, suggestive of a floor effect. Offline gains are characteristic of mirror-reversal tracing [13] as well as motor sequence learning tasks [15, 41, 42]. We found offline gains (reduced % error and crossing points) in the tracing task in both the control and prism groups. In fact, both groups spent very little time tracing outside of the shape’s path or crossing the lines of the shape on day 2; % error and crossing point measures hovered close to zero. It is possible that the relatively small sample size of our study limited our ability to detect group differences. However, group sizes of twelve are typical in these types of studies. In addition, our complementary Bayesian analysis supported the results of the % error measure, though the data were insensitive to detect an effect for the crossing points measure. It is also possible that our measures suffered from somewhat of a floor effect. Nonetheless, both groups demonstrated strong evidence of learning.

Adapting to prisms during a seated pointing task prior to the mirror-reversal tracing task did not interfere with initial learning of this latter task. Some task combinations do cause anterograde interference of acquisition, especially those with similar context but conflicting or opposite mappings (e.g., [9, 37, 38]). The apparent absence of interference in our experiment was likely due to differences in learning between these two tasks; visuomotor adaptation requires updating of an existing control policy whereas mirror-reversal tracing requires learning a new control policy [1, 12–14]. Others have shown that people can learn different motor tasks concurrently. For example, learning novel kinematics (i.e., visuomotor rotations) and novel dynamics (in the form of added mass attached to the arm or reaching with a velocity-dependent force applied to the arm) do not interfere with one another [43, 44]. Alternatively, the presence of a washout block of trials following the prism adaptation (i.e., the post-adaptation trials in our study) could have prevented interference [9].

Some mechanisms of motor learning may inhibit learning a subsequent task, potentially counteracting the effect we sought to test. Long-term potentiation-(LTP)-like plasticity in human motor cortex is transiently reduced, or occluded, following simple motor learning tasks [45–47]. This implies a finite capacity for plasticity for some time after prior learning, which could impair subsequent learning. If this were true in our study, we might find disrupted learning on the tracing task because of the plasticity induced by the prisms. However, both groups learned the tracing task on day 1. In addition, our findings of high day 2 performance and offline gains in the prism group suggest that this effect did not impact retention of our tracing task. We speculate that the lack of LTP-like occlusion effect may relate to different subsets of neurons within primary motor cortex (or other learning-related brain regions) involved in our motor tasks.

Since we performed post-adaptation trials with pointing to the same target location as during prism exposure, the aftereffects present in our results may not reflect true adaptation (that is, we cannot definitively conclude that spatial realignment/internal model updating occurred). However, extensive research [5, 6] strongly supports the notion that exposure to prisms leads to spatial realignment (internal model updating). In addition, we have previously shown that exposure to constant prism shifts during pointing transfers to other tasks and contexts [34] and, using a prism noise protocol, that prism adaptation during walking involves state estimation and updating of an internal model based on sensory prediction error [3]. Perhaps most importantly, there is little debate that exposure to prisms leads to neuroplasticity, the main objective of using this task in the present study. Thus, we do not believe our method to assess prism aftereffects affected the ability to address our research question.

There are several possible reasons why prior prism adaptation may not have enhanced subsequent *de novo* learning, which provide potential avenues for future research. First, the choice of a prism noise protocol rather than a constant prism diopter for the visuomotor perturbation may have influenced our results. Indeed, studies showing that prism adaptation improves symptoms of spatial neglect use a constant prism diopter [48]. However, adaptation occurs more rapidly with this design compared to a noise protocol [3], thus reducing the time the brain must learn the mapping. It is unclear whether there are differences in metrics of plasticity between a noise and constant prism protocol. It is also possible that exposing participants to alternating blocks of trials with large prism shifts in opposite directions would have better facilitated subsequent learning. Second, participants may have required additional prism exposure trials to maximize the priming effect of prisms on neuroplasticity. However, our previous work using a similar number of exposure trials led to generalization to other tasks [7, 34] and consolidation [7, 8, 11]. Other groups have also used 60 trials and found spatial realignment (e.g., [35]). Regardless, we observed a reduction in errors and return to baseline performance within the allotted prism exposure phase, supporting the notion that neuroplasticity occurred. Third, we may not have used the best adaptation task (i.e., pointing with the hand) to optimize the potential of prior prism exposure. There is evidence that adapting to prisms while walking and stepping onto a target leads to stronger consolidation than reaching to a target with the arm [49]. In addition, exposing participants to multiple tasks while adapting to prisms might have led to greater effects on the tracing task. Fourth, the choice of *de novo* learning task might explain the lack of group differences. Although prism adaptation did not enhance subsequent mirror-reversal tracing, it may enhance other motor skills, such as learning how to play an instrument or a certain sports skill. Ultimately, we believe our experimental rationale is sound and thus, these suggested future experiments should be conducted before concluding that prior prism adaptation (or any prior motor learning) is not beneficial to subsequent motor skill learning. Moreover, seeking such an effect is worthwhile because there are advantages to having a cheap, non-invasive, and non-stimulation-based approach to facilitate learning and recovery of function following injury.

## Supporting information

S1 Table Bayesian model comparison and estimates of best fitting model for % error on day 1 learning. BF_10_ = Bayes Factor (where _10_ refers to the alternative hypothesis, *H*_1_, relative to the null hypothesis, *H*_0_); CI = credible intervals. Participant’s random effect included in all models. Best fitting model is bolded.
(PDF)

S2 Table Bayesian model comparison and estimates of best fitting model for completion amount on day 1 learning. BF_10_ = Bayes Factor (where _10_ refers to the alternative hypothesis, *H*_1_, relative to the null hypothesis, *H*_0_); CI = credible intervals. Participant’s random effect included in all models. Best fitting model is bolded.
(PDF)

S3 Table Bayesian model comparison and estimates of best fitting model for crossing points on day 1 learning. BF_10_ = Bayes Factor (where _10_ refers to the alternative hypothesis, *H*_1_, relative to the null hypothesis, *H*_0_); CI = credible intervals. Participant’s random effect included in all models. Best fitting model is bolded.
(PDF)

S4 Table Bayesian model comparison and estimates of best fitting model for % error for the offline gains analysis. BF_10_ = Bayes Factor (where _10_ refers to the alternative hypothesis, *H*_1_, relative to the null hypothesis, *H*_0_); CI = credible intervals. Participant’s random effect included in all models. We found two potential best fitting models. There was weak evidence in favor of the model with main effects of both Day and Group relative to the model with only the main effect of Day. We chose to analyze the more complex model so that we could check for differences between groups. Chosen model is bolded.
(PDF)

S5 Table Bayesian model comparison and estimates of best fitting model for completion amount for the offline gains analysis. BF_10_ = Bayes Factor (where _10_ refers to the alternative hypothesis, *H*_1_, relative to the null hypothesis, *H*_0_); CI = credible intervals. Participant’s random effect included in all models. Best fitting model is bolded.
(PDF)

S6 Table Bayesian model comparison and estimates of best fitting model for crossing points for the offline gains analysis. BF_10_ = Bayes Factor (where _10_ refers to the alternative hypothesis, *H*_1_, relative to the null hypothesis, *H*_0_); CI = credible intervals. Participant’s random effect included in all models. Best fitting model is bolded.
(PDF)
