# Supplementary material for: The effects of prior exposure to prism lenses on de novo motor skill learning
Source: PLoS One. 2023 Oct 20;18(10):e0292518. doi: 10.1371/journal.pone.0292518 (PMC10588867; doi:10.1371/journal.pone.0292518)
Supplement: S1 Table — BF10 = Bayes Factor (where 10 refers to the alternative hypothesis, H1, relative to the null hypothesis, H0); CI = credible intervals. Participant’s random effect included in all models. Best fitting model is bolded. (PDF) [file pone.0292518.s001.pdf]

S1 Table. Bayesian model comparison and estimates of best fitting model for % error on day 1 learning. BF_{10} = Bayes Factor (where $_{10}$ refers to the alternative hypothesis, H_1, relative to the null hypothesis, H_0); CI = credible intervals. Participant's random effect included in all models. Best fitting model is bolded.

Day 1, % Error

Model	BF_{10}
H_0 = base model (random effect: Participant)	-
H_1 = main effect of Bin	1.4e+21
H_1 = main effect of Group	36.1
H_1 = main effects of Bin & Group	5.0e+22
H_1 = main effects (Bin & Group) + interaction	5.7e+26

Model	BF_{10}
H_0 = main effects of Bin & Group	-
**H_1 = main effects (Bin & Group) + interaction**	**1.1e+4**

Parameter (from best fitting model)	Estimate [95% CI]
Intercept	39.9 [30.2, 49.8]
Group[Prism]	-8.0 [-22.7, 6.4]
Bin[Bin2]	-8.4 [-14.3, -2.6]
Bin[Bin3]	-15.0 [-20.6, -9.2]
Bin[Bin4]	-20.2 [-25.9, -14.3]
Bin[Bin5]	-20.9 [-26.6, -15.3]
Group[Prism]Bin[Bin2]	-1.7 [-10.0, 6.6]
Group[Prism]Bin[Bin3]	0.1 [-8.2, 8.5]
Group[Prism]Bin[Bin4]	1.2 [-7.3, 9.5]
Group[Prism]Bin[Bin5]	2.1 [-6.2, 10.4]
