# Supplementary material for: The effects of prior exposure to prism lenses on de novo motor skill learning
Source: PLoS One. 2023 Oct 20;18(10):e0292518. doi: 10.1371/journal.pone.0292518 (PMC10588867; doi:10.1371/journal.pone.0292518)
Supplement: S2 Table — BF10 = Bayes Factor (where 10 refers to the alternative hypothesis, H1, relative to the null hypothesis, H0); CI = credible intervals. Participant’s random effect included in all models. Best fitting model is bolded. (PDF) [file pone.0292518.s002.pdf]

S2 Table. Bayesian model comparison and estimates of best fitting model for completion amount on day 1 learning. BF_{10} = Bayes Factor (where $_{10}$ refers to the alternative hypothesis, H_1, relative to the null hypothesis, H_0); CI = credible intervals. Participant's random effect included in all models. Best fitting model is bolded.

Day 1, Completion Amount

Model	BF_{10}
H_0 = base model (random effect: Participant)	-
H_1 = main effect of Bin	4.0e+25
H_1 = main effect of Group	55.0
H_1 = main effects of Bin & Group	2.2e+27
H_1 = main effects (Bin & Group) + interaction	7.0e+34

Model	BF_{10}
H_0 = main effects of Bin & Group	-
**H_1 = main effects (Bin & Group) + interaction**	**3.2e+7**

Parameter (from best fitting model)	Estimate [95% CI]
Intercept	256.2 [223.1, 289.3]
Group[Prism]	-34.7 [-84.6, 15.8]
Bin[Bin2]	35.9 [6.2, 66.2]
Bin[Bin3]	73.5 [43.8, 103.2]
Bin[Bin4]	89.4 [58.8, 118.8]
Bin[Bin5]	100.4 [69.1, 130.0]
Group[Prism]Bin[Bin2]	30.7 [-11.8, 73.7]
Group[Prism]Bin[Bin3]	26.8 [-16.2, 70.4]
Group[Prism]Bin[Bin4]	35.3 [-8.4, 78.8]
Group[Prism]Bin[Bin5]	35.6 [-8.0, 78.8]
