# Supplementary material for: The effects of prior exposure to prism lenses on de novo motor skill learning
Source: PLoS One. 2023 Oct 20;18(10):e0292518. doi: 10.1371/journal.pone.0292518 (PMC10588867; doi:10.1371/journal.pone.0292518)
Supplement: S3 Table — BF10 = Bayes Factor (where 10 refers to the alternative hypothesis, H1, relative to the null hypothesis, H0); CI = credible intervals. Participant’s random effect included in all models. Best fitting model is bolded. (PDF) [file pone.0292518.s003.pdf]

S3 Table. Bayesian model comparison and estimates of best fitting model for crossing points on day 1 learning. BF_{10} = Bayes Factor (where $_{10}$ refers to the alternative hypothesis, H_1, relative to the null hypothesis, H_0); CI = credible intervals. Participant's random effect included in all models. Best fitting model is bolded.

Day 1, Crossing Points

Model	BF_{10}
H_0 = base model (random effect: Participant)	-
**H_1 = main effect of Bin**	**6.4e+9**
H_1 = main effect of Group	0.116
H_1 = main effects of Bin & Group	7.4e+8
H_1 = main effects (Bin & Group) + interaction	1.4e+5

Model	BF_{10}
H_0 = main effects of Bin & Group	-
H_1 = main effects (Bin & Group) + interaction	0.0002

Parameter (from best fitting model)	Estimate [95% CI]
Intercept	0.29 [0.25, 0.34]
Bin[Bin2]	-0.12 [-0.17, -0.08]
Bin[Bin3]	-0.17 [-0.22, -0.12]
Bin[Bin4]	-0.19 [-0.24, -0.14]
Bin[Bin5]	-0.20 [-0.25, -0.16]
