# Supplementary material for: The effects of prior exposure to prism lenses on de novo motor skill learning
Source: PLoS One. 2023 Oct 20;18(10):e0292518. doi: 10.1371/journal.pone.0292518 (PMC10588867; doi:10.1371/journal.pone.0292518)
Supplement: S4 Table — BF10 = Bayes Factor (where 10 refers to the alternative hypothesis, H1, relative to the null hypothesis, H0); CI = credible intervals. Participant’s random effect included in all models. We found two potential best fitting models. There was weak evidence in favor of the model with main effects of both Day and Group relative to the model with only the main effect of Day. We chose to analyze the more complex model so that we could check for differences between groups. Chosen model is bolded. (PDF) [file pone.0292518.s004.pdf]

S4 Table. Bayesian model comparison and estimates of best fitting model for % error for the offline gains analysis. BF_{10} = Bayes Factor (where $_{10}$ refers to the alternative hypothesis, H_1, relative to the null hypothesis, H_0); CI = credible intervals. Participant's random effect included in all models. We found two potential best fitting models. There was weak evidence in favor of the model with main effects of both Day and Group relative to the model with only the main effect of Day. We chose to analyze the more complex model so that we could check for differences between groups. Chosen model is bolded.

Offline gains, % Error

Model	BF_{10}
H_0 = base model (random effect: Participant)	-
H_1 = main effect of Day	552.3
H_1 = main effect of Group	1.1
H_1 = main effects of Day & Group	531.2
H_1 = main effects (Day & Group) + interaction	347.3
**Model**	**BF_{10}**
H_0 = main effects of Day & Group	-
H_1 = main effects (Day & Group) + interaction	0.65
**Model**	**BF_{10}**
H_0 = main effect of Day	-
**H_1 = main effects of Day & Group**	**1.03**
**Parameter (from chosen model)**	**Estimate [95% CI]**
Intercept	2.48 [1.89, 3.07]
Day[Day2]	-0.43 [-0.61, -0.24]
Group[Prism]	-0.06 [-0.94, 0.83]
