# Supplementary material for: The effects of prior exposure to prism lenses on de novo motor skill learning
Source: PLoS One. 2023 Oct 20;18(10):e0292518. doi: 10.1371/journal.pone.0292518 (PMC10588867; doi:10.1371/journal.pone.0292518)
Supplement: S5 Table — BF10 = Bayes Factor (where 10 refers to the alternative hypothesis, H1, relative to the null hypothesis, H0); CI = credible intervals. Participant’s random effect included in all models. Best fitting model is bolded. (PDF) [file pone.0292518.s005.pdf]

S5 Table. Bayesian model comparison and estimates of best fitting model for completion amount for the offline gains analysis. BF_{10} = Bayes Factor (where $_{10}$ refers to the alternative hypothesis, H_1, relative to the null hypothesis, H_0); CI = credible intervals. Participant's random effect included in all models. Best fitting model is bolded.

Offline gains, Completion Amount

Model	BF_{10}
H_0 = base model (random effect: Participant)	-
H_1 = main effect of Day	15.2
H_1 = main effect of Group	19.2
H_1 = main effects of Day & Group	282.8
H_1 = main effects (Day & Group) + interaction	5558.4
**Model**	$\mathbf{BF_{10}}$
H_0 = main effects of Day & Group	-
$\boldsymbol{H_1}$ **= main effects (Day & Group) + interaction**	**19.5**
**Parameter (from best fitting model)**	**Estimate [95% CI]**
Intercept	355.5 [344.6, 366.6]
Day[Day2]	-4.7 [-15.4, 6.3]
Group[Prism]	1.2 [-15.0, 17.1]
Day[Day2]Group[Prism]	2.2 [-13.5, 17.8]
