# Supplementary material for: The effects of prior exposure to prism lenses on de novo motor skill learning
Source: PLoS One. 2023 Oct 20;18(10):e0292518. doi: 10.1371/journal.pone.0292518 (PMC10588867; doi:10.1371/journal.pone.0292518)
Supplement: S6 Table — BF10 = Bayes Factor (where 10 refers to the alternative hypothesis, H1, relative to the null hypothesis, H0); CI = credible intervals. Participant’s random effect included in all models. Best fitting model is bolded. (PDF) [file pone.0292518.s006.pdf]

S6 Table. Bayesian model comparison and estimates of best fitting model for crossing points for the offline gains analysis. BF_{10} = Bayes Factor (where $_{10}$ refers to the alternative hypothesis, H_1, relative to the null hypothesis, H_0); CI = credible intervals. Participant's random effect included in all models. Best fitting model is bolded.

Offline gains, Crossing Points

Model	BF_{10}
H_0 = base model (random effect: Participant)	-
**H_1 = main effect of Day**	**2.0**
H_1 = main effect of Group	0.072
H_1 = main effects of Day & Group	0.13
H_1 = main effects (Day & Group) + interaction	0.026

Model	BF_{10}
H_0 = main effects of Day & Group	-
H_1 = main effects (Day & Group) + interaction	0.174

Parameter (from best fitting model)	Estimate [95% CI]
Intercept	0.09 [0.06, 0.11]
Day[Day2]	-0.02 [-0.03, -0.01]
